# Transition from hospital to home care: a mixed methods study in light of Meleis’s Theory

**DOI:** 10.1590/0034-7167-2023-0357

**Published:** 2025-03-14

**Authors:** Liasse Monique de Pinho Gama, Karoliny Ruama Carrenho Ribeiro, João Lucas Campos de Oliveira, Maria Antônia Ramos Costa, Aline Marques Acosta, Verusca Soares de Souza

**Affiliations:** I Universidade Federal de mato Grosso do Sul. Campo Grande, Mato Grosso do Sul, Brazil; II Universidade Federal do Rio Grande do Sul. Porto Alegre, Rio Grande do Sul, Brazil; III Universidade Estadual do Paraná. Paranavaí, Paraná, Brazil

**Keywords:** Nursing, Patient Discharge, Nursing Care, Transitional Care, Hospitalization, Enfermería, Alta del Paciente, Atención de Enfermería, Cuidado de Transición, Hospitalización

## Abstract

**Objectives:**

to understand transition from hospitalization to home care from the perspective of patients/family members.

**Methods:**

a mixed methods study of concomitant transformative design, with qualitative weighting. Recruitment took place in a hospital in the Midwest, carried out with patients and family members during home visits between ten and 30 days after hospital discharge, between April and September 2022. The Barthel Index, Care Transitions Measure (CTM-15) and audio-recorded interviews were used. Data were analyzed according to transitions theory and integrated using the fusion strategy.

**Results:**

twenty-six patients and 18 family members participated. The nature of transitions involves new care and readmission. In the facilitating and inhibiting conditions, low income and lack of primary care monitoring stood out, and the response pattern was unsatisfactory (CTM-15 score 59.23).

**Conclusions:**

transition from hospital to home care was unsatisfactory. Nurses and multidisciplinary teams should pay attention to actions that include effective discharge planning.

## RESUMEN

**Objetivos:**

comprender la transición del cuidado de la hospitalización al domicilio desde la perspectiva del paciente/familiares.

**Métodos:**

estudio de métodos mixtos de diseño transformativo concurrente, con ponderación cualitativa. El reclutamiento se llevó a cabo en un hospital del Medio Oeste, realizado con pacientes y familiares durante visitas domiciliarias entre diez y 30 días después del alta hospitalaria, entre abril y septiembre de 2022. Se utilizó el Índice de Barthel, la Care Transitions Measure (CTM-15) y entrevistas grabadas en audio. Los datos fueron analizados contra la teoría de las transiciones y integrados por la estrategia de fusión.

**Resultados:**

participaron 26 pacientes y 18 familiares. La naturaleza de las transiciones implica nueva atención y readmisión. En las condiciones inhibidoras facilitadoras destacaron bajos ingresos y falta de seguimiento de cuidados básicos, y el patrón de respuesta fue insatisfactorio (puntuación CTM-15 59,23).

**Conclusiones:**

la transición de la atención hospitalaria a la domiciliaria fue insatisfactoria. Las enfermeras y el equipo multidisciplinario deben prestar atención a acciones que incluyan una planificación eficaz del alta.

## INTRODUCTION

Care transition can be defined as the time interval that begins with the preparation of individuals for discharge and ends when they are received at the next service. It consists of a set of actions that aim to ensure coordination and continuity of care in the transfer of health system users, between the various services of the care network or between units of the same organization, aiming at the effectiveness of care actions and reduction of complications^(1,2)^.

From the perspective of the health-disease process, transition from hospitalization to home care often includes discharge planning actions, patient and family health education, coordination between health services, communication among teams and post-discharge monitoring, with nurses being the main professionals responsible for coordinating this care^(3-5)^.

Quality of care transitions has been used as one of the components for assessing hospital performance. Detailed and updated analysis of care transition strategies at hospital discharge and return home can increase knowledge about continuity of care and encourage its strengthening^(6)^. In Brazil, however, research on the subject is incipient^(1)^. A study conducted in the emergency department of a hospital in southern Brazil, with 117 patients and 81 caregivers, showed moderate quality of care transition and the need to adopt strategies to improve the discharge process from the emergency room and continuity of care for patients with chronic diseases^(3)^.

When nursing care was assessed in the transition from care for hospitalized older adults to home care, it was observed that the highest rate of non-compliance was the lack of continuing training on care transition and hospital discharge plans. The implementation of educational programs led to an improvement in compliance rates, contributing to improving the transition from care for older adults^(7)^.

Although their participation is included in health policies, and their intention is that they are involved in care transition, the elderly population and the population as a whole have been little involved in the decision-making process in planning their hospital discharge^(8)^. Moreover, discontinuity of care and failures in communication among professionals can contribute to adverse events and lead to unnecessary hospital returns, increasing patient and family suffering/discomfort and contributing to worse health outcomes^(9)^.

Given the role of transition as an indicator of health care performance and patient safety, providing visibility and supporting the discharge planning process can support health institutions and nurses in improving their practices, valuing communication for an effective transition that can be enhanced by the use of theoretical frameworks such as Meleis’s. Entitled “Transition Theory: mid-range and situation-specific theories”, by Afaf Meleis, it recognizes that, throughout life, individuals experience transition situations that range from becoming a mother and/or father to witnessing the death of a close family member, going through the phases of human development, and, at the organizational level, encompasses the transitions from states of complete health to situations of illness^(10)^.

Considering transition as a dynamic and complex process, the interventions inherent to this process must be sensitive from the perspective of those who experience it. Thus, the following guiding question was raised: how does transition from hospitalization to home care occur from the perspective of patients/family members?

## OBJECTIVES

To understand transition from hospitalization to home care from the perspective of patients/family members.

## METHODS

### Ethical aspects

The study followed Resolution 466/2012 and was approved by the *Universidade Federal do Amaro Grosso do Sul* Research Ethics Committee, in which participants’ consent was obtained through signing the Informed Consent Form. It originated from the dissertation entitled “*Transição de cuidados da hospitalization hospitalar ao domicílio: estudo de método misto*”.

### Methodological framework

This is mixed methods research that aims to compare different perspectives in order to develop a more complete understanding of the changes needed by a group through the integration of qualitative and quantitative data^(11)^.

### Methodological procedures

#### Study design

This is mixed methods study of the concomitant transformative strategy, with greater emphasis on the qualitative approach (quan + QUAL). The Mixed Methods Appraisal Tool (MMAT) was used to direct the study construction and guide the methodology^(12)^.

This mixed research design has as its central characteristic the emphasis on a certain theoretical light to guide the methodological choices (quantitative and qualitative) and reach the expected inferences^(11)^. Qualitative weight assignment was defined under the understanding that the narratives expressed portray in greater depth the experience lived by participants in transition from hospital to home.

#### Study setting

The study was conducted in a medium-sized public hospital in a city in northern Mato Grosso do Sul. At the time of the study, the facility had 55 beds exclusively for the Unified Health System, of which 35 beds were allocated to a mixed sector for caring for patients from the medical-surgical clinic, where the study was conducted. It is intended for medical clinic care with a greater number of cases of chronic conditions, in addition to general, orthopedic, elective and emergency surgeries. The hospital carries out care, teaching and research activities. It is important to note that, as reported by the Nursing Directorate when authorizing the study, the hospital has a structured discharge plan completed and delivered by nurses based on issues related to devices, injuries/wounds and four lines for including specific care.

### Participant selection

Study participants were patients and their family members. Patients were included in the study if they were 18 years of age or older and had been discharged from the clinical and surgical inpatient units to their homes. Family members were defined as “a group of individuals linked by a deep emotional bond and a sense of belonging to the group”^(13)^. Therefore, family members were considered to be individuals who share common bonds and those who were recognized by patients as family. The inclusion criteria for family members were whether they had lived with a patient for more than six months, which was considered sufficient time to establish a relationship and become familiar with the home routine. It is worth noting that the instrument and the collection scripts allowed for application to both patients and family members.

### Data collection and organization

Data collection was divided into two stages. In the first, patients and family members were recruited during hospitalization and, in the second, between ten and 30 days after hospital discharge, through home visits (HVs), as recommended by the data collection instrument, which allows its applicability to patients who had recently been discharged: from seven days to six weeks. In this regard, in order to minimize forgetfulness by patients/family members, it was decided to carry out visits within 30 days, within the time frame established by the authors of the instrument^(4)^.

At the time of recruitment at the hospital, patients who lived in Nursing Homes (NH), in rural areas and outside the municipality where the hospital is located were excluded from the study. After recruitment, patients who died and those who, for some reason, after three attempts to schedule and visit, did not perform data collection at home were excluded.

**Figure 1:**
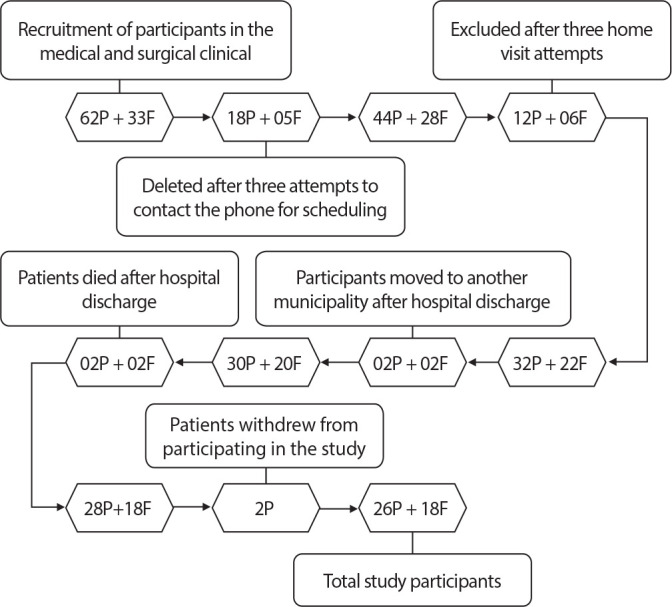
Flowchart of study participants, Coxim, Mato Grosso do Sul, Brazil, 2022

The 62 patients who were hospitalized during the data collection period were recruited and approached for the second stage, as shown in the participant selection flowchart (Figure 1).

*P - patients; F - family members.*

Data were collected between April and September 2022. Patients and family members were approached for convenience, and given the emphasis on the qualitative approach, recruitment continued until data saturation was achieved^(14)^.

Four data collection instruments were used, such as a patient and family characterization form, the Barthel Index and the Care Transitions Measure (CTM-15), for quantitative analysis, and an interview guide for patients and family members, for qualitative analysis.

The Barthel Index, arranged in a scalar format, aims to assess activities of daily living (ADLs) and measure the degree of functional independence of individuals^(15)^. Functional independence assessment was performed at two times: one during patients’ hospitalization period during recruitment and the other during HV. The assessment is made using a score from zero to five points, with zero being described as “total inability to perform the task” and five as “independent without effort”. The ten ADLs assessed are eating, bathing, dressing, using the bathroom, transferring, mobility, going up and down stairs, walking, handling feces and handling urine.

The CTM-15, translated and validated for Brazilian culture, seeks to assess the quality of care transitions from patients’ perspective. It consists of 15 statements divided into four categories, which recognize distinct situations of care transition, from the moment of hospitalization to post-discharge follow-up^(4)^. Composed of 15 items, they are grouped into four domains, such as information transfer, patient and caregiver preparation, support for self-management and empowerment to assert preferences, measured by a Likert-type scale, with five response options (Strongly disagree; Disagree; Agree; Strongly agree; Do not know/do not remember/not applicable).

The interview script was based on the guiding question “How did you plan your (or your family member’s) hospital discharge and transition from hospital to home happen?”, and supporting questions were used to encourage greater participant interaction and speech. It should be noted that, in the case of patient and family participation, the interviews took place with both simultaneously so that one complemented the other’s speech and, therefore, the results are presented together.

### Data analysis and presentation

The data from interviews were recorded and later transcribed, and the excerpts analyzed were based on the Transition Theory. To this end, the interviews were manually read to exhaustion, and their excerpts were grouped based on the theoretical framework, understood through the existence of three basic constructs, namely: nature of transitions; facilitating and inhibiting conditions; and response patterns with process and result indicators^(16)^.

Concerning narrative organization, they were structured in an alphanumeric system, preserving participant anonymity. The letter “P” was used for patients and the letter “F” for family members, with numbering following the chronological order of the interviews (P1; P2; F3; P4; F4; F5). Furthermore, language errors were removed to facilitate understanding participants’ speech without altering the meaning of sentences. The *a priori* analysis was applied to the speeches, which is characterized by the fact that the researcher has, in advance, pre-defined categories. Using Meleis’s theory, the excerpts were categorized using the thematic chunks of the framework and, thus, presented in the results, namely: Nature of transitions; Facilitating and inhibiting conditions of care transition; and Transition response patterns.

Quantitative data were exported to spreadsheets, and descriptive statistics were used to analyze the participants’ profile and functional independence. The Barthel Index was measured by a score ranging from zero to 100 points, verified by the sum of ten categories of ADLs. The higher the score, the greater the person’s independence^(15)^. To analyze the responses to the CTM-15, the instructions of the authors of the original instrument were followed, presenting the results in scores ranging from zero to 100^(17)^. It should be noted that the instrument does not have a cut-off point for classification. However, supported by other investigations that used the same questionnaire, a satisfactory transition was considered for those with scores equal to or greater than 70^(17)^.

After analyzing quantitative and qualitative data, they were integrated using the fusion strategy, which consists of a mixture in which quantitative and qualitative data are grouped through a combined analysis^(18)^. The speaking table strategy was used to present the data^(19)^.

## RESULTS

The sample consisted of 26 patients and 18 family members. Patients were equally composed of 13 men and 13 women, aged between 26 and 89 years (mean: 62; SD 17.51). Most participants self-identified as brown (61.5%), with a elementary education level (46.2%). Half of participants (50%) stated that they were retired and 12 (46.1%) were married. The majority of study participants (84.6%) lived with their families.

Of the 18 family members, 11 (61.1%) were female, aged between 18 and 76 years (mean: 50; SD 16.87). They reported being married (44.4%), employed (61.1%), living with patients for more than ten years (66.7%) and being the main people providing patient care (77.8%).

Care transition analysis based on Meleis’s theory was organized into three thematic chunks: Nature of transitions; Conditions that facilitate and inhibit care transition; and Response patterns of transition.

### Nature of transitions

**Figure 2:**
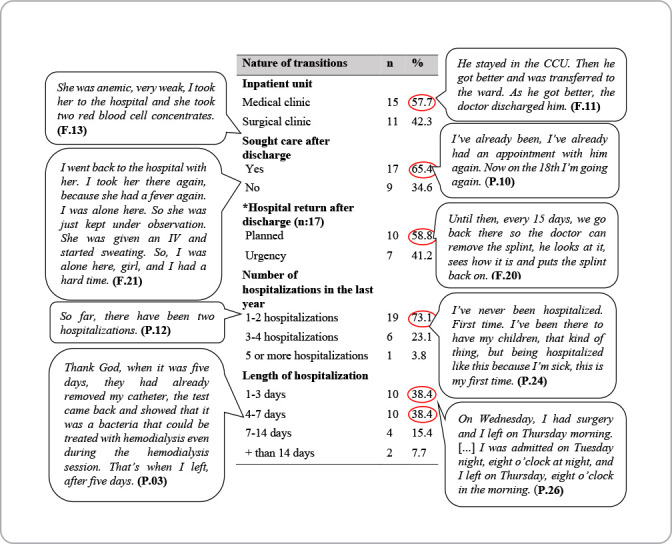
Table showing the nature of transitions of patients discharged from medical or surgical clinics, Coxim, Mato Grosso do Sul, Brazil, 2022

Hospitalizations occurred due to abdominal/digestive system problems (07), respiratory, renal, vascular and bone problems (4, respectively), and endocrine, genital and cancer problems (1, respectively). Considering the above, the following table (Figure 2) shows the nature of the transitions of patients who were discharged from the medical or surgical clinic.

Some participants highlighted factors that implied new care and hospital readmission after transition to home care.

*I got home and I was fine. I was fine for two days. On the third day, I had to go back. Then I was hospitalized for another five days because of a catheter I had that was infected. I had to remove the catheter to stop the infection.* (P. 03)

*I had a bug on my finger, so he admitted me. I stayed there for four and a half days. He discharged me on Sunday at nine o’clock, and I left. On Monday, he told me to go to the clinic here. I went, got there, they treated me, bandaged me, and thought it was really bad. My doctor sent me another referral to the hospital. I was admitted again, and stayed for another four days* [...] *it was bad, it was bad, but even so, I was discharged. She told me to go back to the hospital again, and I stayed for another four days.* (P. 12)

*Because there’s a little hole here* [1^st^ toe], *and it doesn’t hurt at all. I think it’s because of the diabetes. I don’t know if he got a fever.* (P. 22)

*You had fever.* (F. 22)

*So, three times he caught me with a fever. Now I don’t have a fever anymore.* (P. 22)

*On the last day that you were feeling ill, it was your* [husband] *who took you to SAMU, right? It was at night. He was the one who took you, right? I took you the other time. I think I took you three times, but you were kept under observation, and then the last time you took you, you were hospitalized.* **(**F. 21)

[...] *twice, for sure, or more than once, she was hospitalized this year. Twice, I’m sure!* (F. 21)

*Look, in the last 12 months, she must have been hospitalized five or six times. Hospitalized, in quotes, because, like, she went to the hospital, because sometimes she stayed there, spent the day in the emergency room and went home, you know? Hospitalized, in the long term, it was three times.* (F. 25)

From another angle, they observed that transition planning is an active process and that, even though a forecast of discharge was guided, daily assessments brought changes.

*The doctor decided that the two bags of blood and she was getting better would be a good idea to discharge her. Also, staying in the hospital for a long time, as she is already old and has other comorbidities, is also a risk.* (F. 13)

*The first time, I found out it was for seven days* [hospitalization period]. *As it got better during treatment, it got better, and the doctor said, “It’s not for three days”. And I was admitted on Wednesday and left on Friday.* (P. 24)

### Conditions that facilitate and inhibit care transition

**Figure 3:**
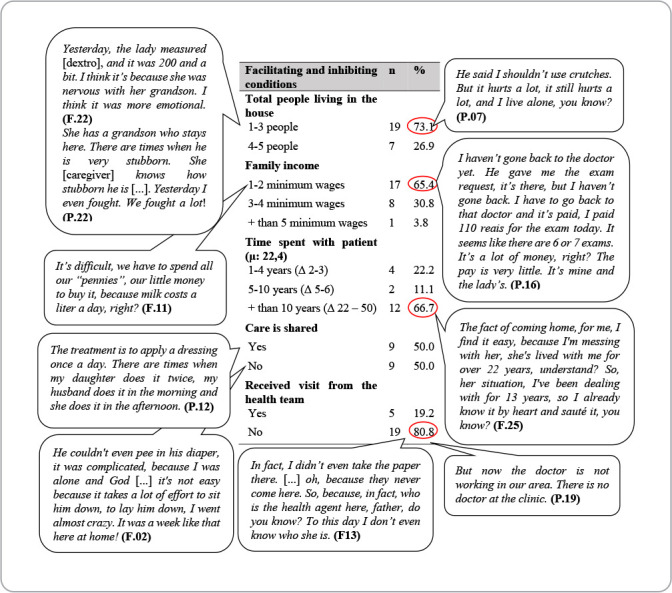
Table of facilitating and inhibiting conditions of transitions of patients discharged from medical or surgical clinics, Coxim, Mato Grosso do Sul, Brazil, 2022

Some factors stood out as challenges to discharge transition, especially family dynamics and income. Figure 3 presents these data.

Some patients, in addition to family arrangements for organizing care during the convalescence period, had to move around the homes of their family and friends.

*For her, it’s better for me to be here. It’s harder for me to be there, and for her to be here. And it’s easier for her to be here. When I get better, I’ll go back to my little ranch.* (P. 10)

*I went to my friend’s house, who was with me, and she didn’t want to leave me alone, because it’s just me and my brother. My brother doesn’t stop. So, she said, “Let’s go to your house, get your things and let’s go to my house”. I stayed there with her for 14 days, until I recovered a little and could come home.* (P. 14)

Low income stood out among the inhibiting conditions for care transition.

*He needs some help from the government, because how is he going to pay the rent, the water, the electricity, how is he going to support himself here? And he is out of work, because he has no way to work.* (F. 07)

*This ointment costs 114 reais. Hydrogel is not a cheap ointment. It’s full. I’ve already used it twice.* (P. 10)

*I have a lady who has been treating me for many years, and I have to work a lot, I’m running out of money. I can’t handle it, everything is private, almost everything is private. She’s seeing, over the years, a CT scan costs 750, one day 800, 900 reais, one day 1.300, 1.200.* (F. 21)

Monitoring after transition to home care, by Primary Care, divided participants’ opinions.

*Until today, no one has come to visit my house, but this week I went there and they said they would come, that the doctor and the nurse would come from this week, but they didn’t come.* (F. 02)

*The health center here takes a long time to make an appointment. The doctor there gave me a referral to go to the health center, to make an appointment there.* [...] *the only person who came here was the girl to bring that referral* [...] *the health worker, only her. But she said that if I have any questions that I can’t go there, they’ll come here, the nurse with the doctor. But they haven’t arrived yet, no* (P. 12)

*No. So far no one* [BHU team] has *come.* (P. 23)

*Geez, it’s hard for anyone to get through, only CHW.* (F. 23)

*I received a visit from the doctor, the nurse and my health worker at the health center.* (P. 15)

*And we have this dignity, this respect to speak, because before I was the one who was taking care of her, it was just me, just me and God, there were no companions* [caregiver]. *The doctor was here, I think twice, once I wasn’t there, he came here, the girl from there who works, how do you say?* [community health worker]. *The nurse, the* [nursing technician] *came too.* (F. 21)

### Transition response patterns

**Figure 4:**
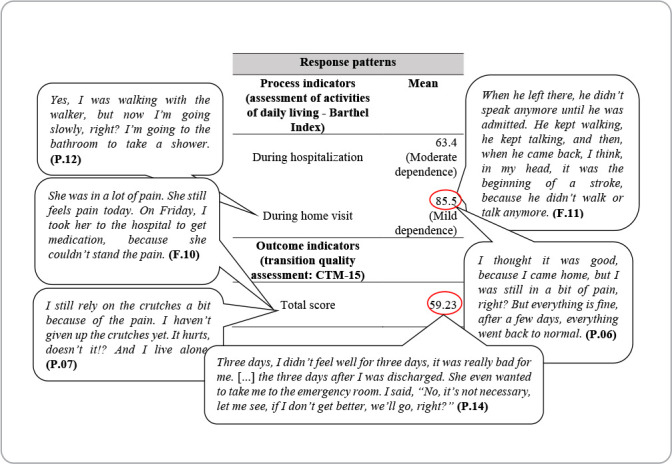
Table of response patterns of transitions of patients discharged from medical or surgical clinics, Coxim, Mato Grosso do Sul, Brazil, 2022

A decrease in the mean dependence for performing ADLs was observed after hospital discharge when compared with the period in which patients were hospitalized, evolving from moderate dependence to mild dependence, as demonstrated in Figure 4.

Patients and family members associated the process of transition from hospital to home care and the evolution of their health status with the ability to walk and perform ADL.

*When she was discharged, she wasn’t walking. The physiotherapist said she should be able to walk, but now she can walk and everything. That’s when she had the catheter removed, after she had the catheter removed, she started walking.* (F. 19)

[...] *I stood up with the walker. Then I took a couple of steps, by myself, holding on. Then I started slowly, walking from there to here, then I soon started to let go of the walker and started walking. It was like this.* [...] *I go to the bathroom, turn on and off the air conditioning, sit on the bed, when I’m not sleepy, I clean the house too. Sometimes, I watch TV.* (P. 22)

*He came here by ambulance, it wasn’t easy, because he couldn’t stand up, sit down or walk, he was completely immobilized, he couldn’t even sit up on his own* [...] *then, he arrived here at home, 11 days later, I didn’t know what to do, because you see a person who walks, and suddenly, he’s not walking, he’s not sitting, he’s not doing anything, he’s not eating, it was really complicated!* (F. 02)

Other patients considered that they were not fit for hospital discharge, and there was no pain management, which generated complaints and discomfort, according to the reports below.

*She was in a lot of pain. She still feels pain today. On Friday, I took her to the hospital to take medication, because she couldn’t stand the pain.* (F. 10)

*I left with pain, but they discharged me anyway. I came back again, and then I went there, took the serum with the medication, and got better. But you can’t put that ointment on me, that ointment drives me crazy. I get here, and the pain lasts all day, all night. Woman, I can’t stand it. How am I going to put up with pain like that day and night?* (P. 10)

*I didn’t feel well for three days, no, it was really bad for me.* [...] *The three days after I was discharged. She even wanted to take me to the emergency room, I said, “No, it’s not necessary, let me see, if I don’t get better, we’ll go”.* (P. 14)

*So, I was even worried, you know? Because, like, all the time, she was very sleepy, you know?* [...] *and then, they took the tube out and took her out so she could go home. And then, she continued the treatment at home. Do you know what it’s like when someone sleeps 24 hours a day? That slow person, who wouldn’t open her eyes, wouldn’t talk, you know? She wasn’t reacting, right? She slept a lot all the time. So much so that the medication was stopped on Thursday, okay? She started reacting last night.* (F. 25)

**Figure 5:**
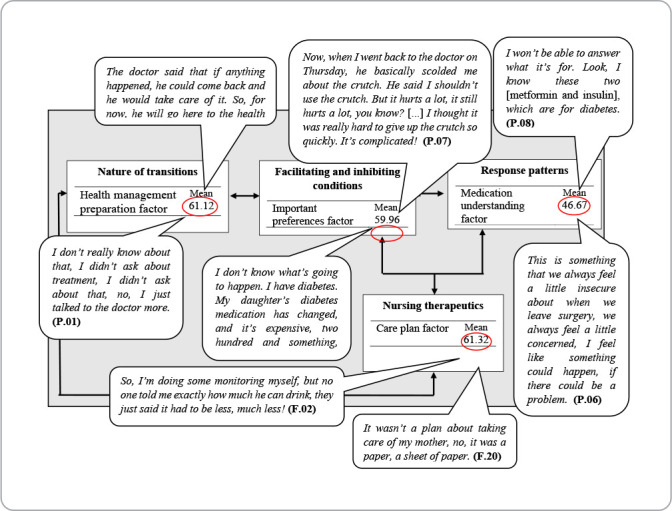
Speaking table of the diagram representing the transitions process, Coxim, Mato Grosso do Sul, Brazil, 2022

Participants’ narratives converge with the results of the questions addressed in CTM-15, and can be better visualized in Figure 5. The figure represents a demonstration of the basic constructs of the theory, organized into four CTM-15 factors, namely: Health management preparation; Medication understanding; Important preferences; and Care plan.

## DISCUSSION

Transition from hospitalization to home care was influenced by several factors. As for the nature of transitions, reports of return after hospital discharge stood out, in which, of the 65.4% of participants who sought health care after hospital discharge, 41.2% reported seeking emergency care. As illustrated in the narratives above, the unscheduled return to the emergency service with the same clinical complaints requires responsibility, development and implementation of an effective, safe and responsible discharge plan. In addition to this, it generates an epidemiological alert that should be seen as an indicator of health service performance^(20,21)^.

In order to avoid or at least minimize the need for emergency care and readmissions after hospital discharge, discharge planning should aim to identify and define individuals’ needs within the context of discharge from the hospital to the community, with a close eye on care transition. To this end, practices are needed to improve hospital discharge management in order to ensure continuity of patient care^(22,23)^.

The various activities performed by nurses during discharge plan can be distinguished among health establishments. Intending to develop and validate a list of activities to be performed by nurses at hospital discharge, a methodological study achieved the validation of 13 of the 14 activities for responsible hospital discharge, contributing to the safe discharge process and resulting in an improvement in the quality of care provided to patients^(24)^.

It is important to highlight that the discharge plan implemented in the service investigated is limited to issues of surveillance of infections related to healthcare and, therefore, does not meet patients’ health needs, which is reinforced by patients’ report that they do not acknowledge having received a care plan. Although the care plan factor was the best assessed in this investigation, perhaps justified by the use of the aforementioned discharge plan, none of the factors received a satisfactory evaluation with a score higher than 70 points.

A review study with randomized clinical trials demonstrated that a structured and personalized discharge plan for individuals provides a decrease in the rates of unplanned readmission for older adults and also increases the chances of reducing length of hospital stay, which could free up capacity for subsequent admissions in a system where there is a shortage of hospital beds^(25)^. Hence, the implementation of effective, responsible and safe discharge planning in the hospital would not only minimize the demand for emergency care after hospital discharge, but would also reduce patient length of hospital stay.

Family dynamics and financial conditions are factors that influenced care transition, reinforcing the importance of knowing the socioeconomic and cultural reality of the population that uses health services, since 65.4% of participants in this study had a family income of up to two minimum wages. A study conducted with 289 older adults in a medium-sized city in the countryside of São Paulo analyzed private spending on the purchase of medications, and family income commitment indicated that 68.9% of participants had economic classification type C (mean family income R$ 1,740.00 - R$ 3,085.00 (Brazilian currency)), according to the *Critério de Classificação Econômica Brasil* (CCEB, Brazilian Economic Classification Criteria). Therefore, in some cases, these expenses became harmful to the family budget^(26)^.

Among the determinants of health, the low income of participants is a condition that inhibits the healthy transition process, being an aggravating factor for access to and cost of medications, as verified in participants’ statements. Medication acquisition is one of the ways in which inequality can manifest itself in society. The expansion of the free provision of medications would be necessary to expand access and avoid expenses^(26)^. In addition, another study supports the statement that public health system users have difficulty completing and/or adhering to treatment due to the lack of medications in the units; consequently, the presence of a pharmacist in Family Health Units can guarantee greater access to medications for users as well as rational use^(27)^. The high costs of prescribing medications not covered by the Unified Health System and requested tests can encourage treatment abandonment, resulting in returns to the emergency service and an unhealthy transition.

Care transition has become one of the domains interconnected with the principles of health systems integration^(28)^. In this regard, Primary Health Care (PHC) is a facilitating resource for the transition process, in addition to being considered the main strategy for reorienting the care model, indicated as a communication center for networks, promoting equity, access, quality of health care and continuity of care provided^(29)^. However, a significant portion (80.8%) of participants reported that they did not receive a HV from any member of the PHC health team. There is a lack of communication between the city’s care networks as well as a counter-referral document, which reflects a gap in care transition.

Failure to perform HVs was a finding of a study that aimed to identify the prevalence of non-performance of HVs by community health workers (CHWs) and associated factors, demonstrating that 18.6% of families were not visited. The aforementioned investigation showed that 52.0% of users said that the CHW in the area did not perform HVs; 26.0% did not know of the existence of CHWs in the neighborhood or unit; and 10.9% reported that, during CHWs’ working hours, there was no one at home to assist them^(30)^. Thus, another study addressed that HVs are health monitoring strategies and facilitators of healthy transition, proving to be efficient in care management, allowing the creation of bonds between families and professionals and increasing the chances of longitudinal care^(28)^.

The process and outcome indicators of care transition are response patterns to be assessed. The usual care provided during the hospitalization period promoted a decrease in dependence to perform ADLs after hospital discharge. This data suggests that, during participants’ hospitalization period, the care provided helps them return to autonomy in daily life activities after hospital discharge, and this is an important issue to be observed during hospitalization and in preparation for discharge from the inpatient unit to home.

Another response pattern to be observed is transition performance. The mean CTM-15 score in this study was 59.2 points, far from being considered satisfactory. A study that used the CTM-15 in southern Brazil involved the participation of 117 patients and 81 caregivers, and assessed the quality of care transition for patients with chronic non-communicable diseases upon discharge from the emergency department to their homes via telephone contact. The mean CTM-15 score was 69.5 points, indicating moderate quality^(4)^.

The quality of inpatient service transition is expected to be better when compared to the emergency service, since patient length of stay is longer, which provides professionals in inpatient sectors with more opportunities to prepare patients for discharge^(4)^. However, the result of the CTM-15 obtained in this study, in particular, compared with in-person interviews at home, brought a better understanding of the factors that make it difficult to achieve adequate response standards, highlighting the disregard of income and socioeconomic conditions, lack of monitoring at other levels of care, difficulty in understanding the guidelines and feeling of unreadiness for discharge.

In this scenario of multiple needs and, often, scarce resources, nurses play a central role in ensuring continuity of treatment and patient safety in self-care, leading and coordinating this process. In this way, nurses act as a mediator in interprofessional actions, paying attention patients/family members’ needs and concerns^(22,24)^.

Nursing is an independent profession that implements and executes interventions related to the care process. Therefore, nurses, since their training, are equipped to carry out health education actions and prescribe care that are part of safe discharge planning. In turn, hospital discharge planning is the responsibility of the multidisciplinary team; however, nurses play a fundamental role in identifying patients’ needs, educating them and their families and in coordinating with the Health Care Network, strengthening continuity of care after hospital discharge^(7)^.

Nurses and the multidisciplinary team must pay attention to actions that include effective, safe and responsible discharge planning in order to minimize the search for emergency services immediately after hospital discharge and promote better results for recovery of well-being and quality of life after discharge.

### Study limitations

The main limitation is related to the type of participant, since the study proposed to analyze care transition for patients admitted to hospital to their homes, and took into account the perspective of patients and family members, excluding professionals, who are essential in the transition process. Therefore, it is suggested that future studies be expanded to include other members involved in the transition process, with a larger sample size of patients and family members.

### Contributions to nursing

The study contributes to nursing by analyzing the performance of care transition from the inpatient sector to home care, according to patients’ and family members’ experience, suggesting a review/reflection of the discharge planning instituted by hospital establishments, in order to support the implementation for better performance of actions aimed at planning hospital discharge.

## CONCLUSIONS

Care transition analysis, using Meleis’s theoretical construct, showed that transition from hospital to home care is far from being considered satisfactory. This interpretation involves several factors, including: socioeconomic and cultural condition of patients and their families; lack of communication with the Health Care Network; feeling of unreadiness for discharge by patients and families; and difficulty in understanding the guidelines provided by the team. These factors directly interfere in achieving a satisfactory transition.
