# Supplementary material for: Transition from hospital to home care: a mixed methods study in light of Meleis’s Theory
Source: Rev Bras Enferm. 2025 Mar 14;78(1):e20230357. doi: 10.1590/0034-7167-2023-0357 (PMC11913045; doi:10.1590/0034-7167-2023-0357)
Supplement: Translation

# Translation: Transição de cuidados hospital-domicílio: estudo de métodos mistos à luz da Teoria de Meleis

**DOI:** 10.1590/0034-7167-2023-0357pt

**Authors:** Liasse Monique de Pinho Gama, Karoliny Ruama Carrenho Ribeiro, João Lucas Campos de Oliveira, Maria Antônia Ramos Costa, Aline Marques Acosta, Verusca Soares de Souza

**Affiliations:** I Universidade Federal de mato Grosso do Sul. Campo Grande, Mato Grosso do Sul, Brasil; II Universidade Federal do Rio Grande do Sul. Porto Alegre, Rio Grande do Sul, Brasil; III Universidade Estadual do Paraná. Paranavaí, Paraná, Brasil

**Keywords:** Enfermagem, Alta do Paciente, Cuidados de Enfermagem, Cuidado Transicional, Hospitalização

## Abstract

**Objetivos:**

compreender a transição do cuidado da internação ao domicílio na perspectiva de pacientes/familiares.

**Métodos:**

estudo de métodos mistos do desenho transformador concomitante, com atribuição de peso qualitativa. O recrutamento aconteceu em hospital do centro-oeste, realizado com pacientes e familiares em visita domiciliar entre dez e 30 dias após a alta hospitalar, entre abril e setembro de 2022. Utilizaram-se o Índice de Barthel, *Care Transitions Measure* (CTM-15) e entrevistas audiogravadas. Os dados foram analisados face à teoria das transições e integrados pela estratégia de fusão.

**Resultados:**

participaram 26 pacientes e 18 familiares. A natureza das transições perpassa por novos atendimentos e reinternação. Nas condições facilitadoras inibidoras, destacaram-se baixa renda e falta de acompanhamento da atenção básica, e o padrão de resposta foi insatisfatório (escore CTM-15 59,23).

**Conclusões:**

a transição do cuidado hospital-domicílio foi insatisfatória. Enfermeiros e equipe multidisciplinar devem se atentar para ações que contemplem um planejamento de alta eficaz.

## INTRODUÇÃO

A transição do cuidado pode ser definida como o intervalo de tempo que se inicia com a preparação do indivíduo para alta, finalizando quando ele é recebido no próximo serviço. Consiste em um conjunto de ações que têm como objetivo assegurar a coordenação e continuidade dos cuidados na transferência de usuários do sistema de saúde, entre os diversos serviços da rede de atenção ou entre unidades de uma mesma organização, visando à efetividade de ações assistenciais e redução de complicações^(1,2)^.

Sob a ótica do processo saúde-doença, a transição do cuidado da internação ao domicílio frequentemente inclui ações de planejamento de alta, educação em saúde do paciente e da família, articulação entre os serviços de saúde, comunicação entre as equipes e acompanhamento pós-alta, sendo os enfermeiros os principais profissionais responsáveis pela coordenação desse cuidado^(3-5)^.

A qualidade das transições do cuidado tem sido utilizada como um dos componentes para avaliação de desempenho de hospitais. A análise detalhada e atualizada das estratégias de transição do cuidado na alta hospitalar e retorno ao domicílio podem aumentar o conhecimento acerca da continuidade do cuidado e estimular seu fortalecimento^(6)^. No Brasil, entretanto, investigações sobre o tema são incipientes^(1)^. Estudo realizado em serviço de emergência de um hospital no sul do país, com 117 pacientes e 81 cuidadores, evidenciou qualidade moderada da transição do cuidado e necessidade de adoção de estratégias para melhorar o processo de alta da emergência e continuidade do cuidado a portadores de doenças crônicas^(3)^.

Quando avaliada a assistência de enfermagem na transição do cuidado à pessoa idosa hospitalizada para o domicílio, observou-se que o maior índice de não conformidade foi a ausência de formação continuada sobre a transição do cuidado e plano de alta hospitalar. A implementação de programas educativos proporcionou melhora nos índices de conformidade, contribuindo para a melhora da transição do cuidado à pessoa idosa^(7)^.

Embora a participação da própria pessoa esteja incluída em políticas de saúde, e sua intenção é que esteja envolvida na transição de cuidados, a população idosa e a população como um todo têm sido pouco envolvidas no processo de tomada de decisão no planejamento de sua alta hospitalar^(8)^. Somado a isso, a descontinuidade do cuidado e as falhas na comunicação entre os profissionais podem contribuir para eventos adversos e ocasionar retornos hospitalares desnecessários, aumentando o sofrimento/desconforto do paciente e da família e contribuindo para piores resultados de saúde^(9)^.

Diante do papel da transição enquanto indicador de desempenho da assistência em saúde e segurança do paciente, dar visibilidade e fundamentar o processo de planejamento de alta pode subsidiar instituições de saúde e enfermeiros na melhoria de suas práticas, valorizando a comunicação para uma transição efetiva que pode ser potencializada pela utilização de referenciais teóricos como o de Meleis. Intitulada “Teoria das Transições: teorias de médio alcance e específicas da situação”, de Afaf Meleis, reconhece que, durante a vida, os indivíduos vivenciam situações de transições que vão desde tornar-se mãe e/ou pai até presenciar a morte de um familiar próximo, percorrendo pelas fases do desenvolvimento humano, e, ao nível organizacional, engloba as transições de estados da saúde íntegra para situações de doença^(10)^.

Assumindo a transição como processo dinâmico e complexo, as intervenções inerentes a este processo devem ser sensíveis a partir da perspectiva de quem a experiencia. Dessa forma, levantou-se a seguinte questão norteadora: como ocorre a transição do cuidado da internação ao domicílio na perspectiva do paciente/familiar?

## OBJETIVOS

Compreender a transição do cuidado da internação ao domicílio na perspectiva de pacientes/familiares.

## MÉTODOS

### Aspectos éticos

O estudo seguiu a Resolução n^o^ 466/2012, e foi aprovado pelo Comitê de Ética em Pesquisa Envolvendo Seres Humanos da Universidade Federal de Mato Grosso do Sul, na qual o consentimento dos participantes ocorreu por meio do Termo de Consentimento Livre e Esclarecido. Originou-se da dissertação intitulada “Transição de cuidados da internação hospitalar ao domicílio: estudo de método misto”.

### Referencial metodológico

Pesquisa de métodos mistos que tem como objetivo comparar diferentes perspectivas, de forma a desenvolver um entendimento mais completo das mudanças necessárias para um grupo por meio da integração dos dados qualitativos e quantitativos^(11)^.

### Procedimentos metodológicos

#### Tipo de estudo

Estudo de métodos mistos da estratégia transformadora concomitante, com maior ênfase na abordagem qualitativa (quan + QUAL). Utilizou-se a ferramenta *Mixed Methods Appraisal Tool* (MMAT) para direcionar a construção do estudo e orientar a metodologia^(12)^.

Este desenho de pesquisa mista tem como característica central dar ênfase a uma determinada luz teórica para guiar as escolhas metodológicas (quantitativas e qualitativas) e alcançar as inferências esperadas^(11)^. A atribuição de peso qualitativo foi definida sob o entendimento de que as narrativas expressadas retratam com mais profundidade a experiência vivenciada pelos participantes na transição do hospital para o domicílio.

#### Cenário do estudo

O estudo ocorreu em hospital público de médio porte de um município do norte do estado de Mato Grosso do Sul. O estabelecimento possuía, no momento da pesquisa, 55 leitos de internação exclusivos do Sistema Único de Saúde, sendo 35 leitos destinados a um setor misto, destinado ao atendimento de pacientes da clínica médica e cirúrgica, onde foi realizado o estudo. Destina-se ao atendimento de clínica médica com maior número de atendimentos a condições crônicas, além de cirurgia geral, ortopédica, eletiva e de emergência. No hospital, são desenvolvidas atividades de assistência, ensino e pesquisa. Cumpre destacar que, conforme informado pela Direção de Enfermagem no ato da autorização da pesquisa, o hospital possui um plano de alta estruturado preenchido e entregue por enfermeiros pautado em questões relacionadas aos dispositivos, lesões/feridas e quatro linhas para inclusão de cuidados específicos.

#### Seleção dos participantes

Os participantes da pesquisa foram pacientes e seus familiares. Para pacientes, incluíram-se aqueles com idade igual ou superior a 18 anos e que tiveram alta das unidades de internação clínica e cirúrgica para o domicílio. Para familiares, adotou-se a definição de família “como um grupo de indivíduos vinculados por uma ligação emotiva profunda e por um sentimento de pertença ao grupo”^(13)^. Logo, considerou-se como família indivíduos que compartilham elos comuns e aqueles legitimados pelo paciente como família. Como critério de inclusão do familiar, levou-se em conta se convivia/residia com o paciente há mais de seis meses, tempo este considerado suficiente para estabelecer uma relação e conhecer a rotina do lar. Vale ressaltar que o instrumento e os roteiros de coleta possibilitaram a aplicação tanto ao paciente quanto ao familiar.

#### Coleta e organização dos dados

A coleta de dados foi dividida em duas etapas. Na primeira, os pacientes e familiares foram recrutados durante a internação hospitalar e, na segunda, entre dez e 30 dias da alta hospitalar, através de visita domiciliar (VD), conforme recomendação do instrumento de coleta de dados, que possibilita sua aplicabilidade com pacientes que tiveram alta recente: de sete dias a seis semanas. Nesse sentido, de forma a minimizar esquecimentos por parte do paciente/familiar, optou-se por realizar as visitas em até 30 dias, dentro do prazo estabelecido pelos autores do instrumento^(4)^.

No momento do recrutamento no hospital, foram excluídos do estudo pacientes que residiam em Instituições de Longa Permanência (ILP), em zona rural e fora do município em que o hospital é localizado. Após o recrutamento, excluíram-se pacientes que evoluíram para óbito e aqueles que, por algum motivo, após três tentativas de agendamento e visita, não realizaram a coleta de dados em domicílio.

Foram recrutados os 62 pacientes que estavam internados no período de coleta de dados, abordados para a segunda etapa, conforme apresentado no fluxograma de seleção dos participantes (Figura 1).

**Figura 1:** Fluxograma dos participantes do estudo, Coxim, Mato Grosso do Sul, Brasil, 2022

*P - pacientes; F - familiares.*

Os dados foram coletados entre os meses de abril e setembro de 2022. A abordagem dos pacientes e familiares realizou-se por conveniência, e, diante da atribuição de ênfase à abordagem qualitativa, o recrutamento se manteve até a obtenção da saturação dos dados^(14)^.

Foram utilizados quatro instrumentos de coleta de dados, como formulário de caracterização de pacientes e familiares, Índice de Barthel e *Care Transitions Measure* (CTM-15), para análise quantitativa, e roteiro de entrevista de pacientes e familiares, para análise qualitativa.

O Índice de Barthel, disposto no formato escalonar, tem como objetivo avaliar as atividades da vida diária (AVDs) e mensurar o grau de independência funcional do indivíduo^(15)^. A avaliação da independência funcional foi realizada em dois momentos: um no período de internação do paciente durante o recrutamento e o outro durante a VD. A avaliação ocorre por meio de pontuação, de zero a cinco pontos, sendo zero descrito como “incapacidade total de realizar a tarefa”, e cinco, “independente sem esforço”. As dez AVDs avaliadas são alimentação, banho, vestir-se, uso do banheiro, transferência, mobilidade, subir e descer escadas, caminhar, manuseio de fezes e manuseio de urina.

O CTM-15, traduzido e validado à cultura brasileira, busca avaliar a qualidade das transições do cuidado a partir da perspectiva do paciente. Tratam-se de 15 afirmações divididas em quatro categorias, as quais reconhecem situações distintas da transição do cuidado, desde o momento da internação até o acompanhamento pós-alta^(4)^. Composto por 15 itens, são agrupados em quatro domínios, como transferência de informação, preparação do paciente, familiar e/ou cuidador, apoio para o autogerenciamento e empoderamento para assegurar preferências, aferidos por escala Likert, com cinco opções de respostas (Discordo muito; Discordo; Concordo; Concordo muito; Não sei/não me lembro/não se aplica).

O roteiro de entrevista foi pautado na questão norteadora “De que forma aconteceram o planejamento da sua alta hospitalar (ou de seu familiar) e a transição do hospital para casa?”, e perguntas de apoio foram empregadas para favorecer maior interação e fala dos participantes. Frisa-se que, no caso de participação do paciente e família, as entrevistas aconteceram com os dois, simultaneamente, de forma que um complementava a fala do outro e, assim, os resultados estão apresentados juntos.

#### Análise e apresentação dos dados

Os dados provenientes das entrevistas foram gravados e posteriormente transcritos, e os excertos analisados foram pautados na Teoria das Transições. Para isso, de forma manual, as entrevistas foram lidas à exaustão, e seus trechos foram agrupados pautando-se no referencial teórico, compreendidas por meio da existência de três construtos basilares, a referir: a natureza das transições; condições facilitadoras e inibidoras; e padrões de resposta com indicadores de processo e resultados^(16)^.

Quanto à organização das narrativas, foram estruturadas em sistema alfanumérico, preservando o anonimato dos participantes. Usou-se a letra “P” para pacientes e a letra “F” para familiares, com numeração seguindo a ordem cronológica das entrevistas (P1; P2; F3; P4; F4; F5). Ademais, vícios de linguagem foram retirados para favorecer o entendimento da fala dos participantes sem alterar o sentido das frases. Aos discursos, foi aplicada a análise apriorística, que se caracteriza pelo fato de o pesquisador possuir, de antemão, categorias pré-definidas. Ao utilizar a teoria de Meleis, os excertos foram categorizados utilizando os blocos temáticos do referencial e, assim, apresentados nos resultados, a saber: Natureza das transições; Condições facilitadoras e inibidoras da transição do cuidado; e Padrões de resposta da transição.

Os dados quantitativos foram exportados para planilhas eletrônicas e, para análise do perfil dos participantes e independência da funcionalidade, utilizou-se estatística descritiva. O Índice de Barthel foi aferido por uma pontuação que varia de zero a 100 pontos, verificada pela somatória de dez categorias de AVDs. Quanto maior a pontuação, maior a independência da pessoa^(15)^. Para a análise das respostas do CTM-15, respeitaram-se as instruções dos autores do instrumento original, apresentando seus resultados em escore que varia de zero a 100^(17)^. Realça-se que o instrumento não apresenta ponto de corte para classificação. Entretanto, amparado por outras investigações que utilizaram o mesmo questionário, considerou-se uma transição satisfatória àquelas com escores iguais ou superiores a 70^(17)^.

Após a análise dos dados quantitativos e qualitativos, realizou-se a integração deles pela estratégia de fusão, que consiste em uma mistura, em que os dados quantitativos e qualitativos são agrupados por meio de uma análise combinada^(18)^. Na apresentação dos dados, empregou-se como estratégia a tabela falante^(19)^.

## RESULTADOS

A amostra foi composta por 26 pacientes e 18 familiares. Os pacientes foram, paritariamente, 13 homens e 13 mulheres, com idade entre 26 e 89 anos (média: 62; DP 17,51). A maioria dos participantes autodeclarou-se parda (61,5%), com grau de escolaridade em nível fundamental (46,2%). Metade dos participantes (50%) afirmou ser aposentada e, 12 (46,1%), casados. Os participantes da pesquisa majoritariamente (84,6%) residiam com seus familiares.

Em relação aos 18 familiares, 11 (61,1%) eram do sexo feminino, com idade entre 18 e 76 anos (média: 50; DP 16,87). Declararam ser casados (44,4%), com vínculo empregatício (61,1%), residindo com o paciente há mais de dez anos (66,7%) e sendo as principais pessoas a desempenharem os cuidados com o paciente (77,8%).

A análise da transição do cuidado pautada na teoria de Meleis foi organizada em três blocos temáticos: Natureza das transições; Condições facilitadoras e inibidoras da transição do cuidado; e Padrões de resposta da transição.

### Natureza das transições

As internações aconteceram por agravos abdominais/sistema digestório (07), do sistema respiratório, renal, vascular e ósseo (4, respectivamente), e do sistema endócrino, genital e câncer (1, respectivamente). Diante do exposto, na sequência, apresenta-se a tabela falante (Figura 2) da natureza das transições dos pacientes que tiveram alta da clínica médica ou cirúrgica.

**Figura 2:** Tabela falante da natureza das transições dos pacientes que tiveram alta da clínica médica ou cirúrgica, Coxim, Mato Grosso do Sul, Brasil, 2022

Alguns participantes destacaram fatores que implicaram novos atendimentos e reinternação hospitalar após a transição para o domicílio.

*Cheguei aqui em casa, eu fiquei bem, dois dias eu fiquei bem. Quando foi no terceiro dia, eu tive que voltar. Aí fiquei mais cinco dias internada por causa de um cateter que eu tinha, que tinha infecção, eu tinha que tirar esse cateter para parar a infecção.* (P. 03)

*Tinha dado um bicho na cabeça do dedo, aí ele me internou. Eu fiquei lá, quatro dias e meio. Ele me deu alta no domingo, às nove horas, eu vim embora. Na segunda-feira, ele mandou eu ir no posto aqui. Eu fui, cheguei lá, fizeram o tratamento, o curativo e achou que estava muito feio. A minha médica mandou outro encaminhamento pra mim ir pro hospital. Eu internei de novo, fiquei mais quatro dias* [...] *estava ruim, estava feio, mesmo assim passou alta. Ela mandou eu voltar de novo pro hospital pra lá, eu fiquei mais quatro dias.* (P. 12)

*Porque aqui tem um furinho* [1° pododáctilo], *e não dói nada. Acho que é por causa da diabetes. Não sei se deu febre.* (P. 22)

*A senhora deu febre, sim.* (F. 22)

*Então, três vezes ele me pegou com febre. Agora não deu mais febre.* (P. 22)

*No último dia que a senhora ficou ruim, foi seu* [marido] *que levou com o SAMU, né? Foi à noite. Foi ele que levou, tá certo? Eu levei na outra vez. Acho que, umas três vezes, eu levei, mas a senhora ficou na observação, aí, nessa última que levou, que internou ela.* (F. 21)

[...] *por duas vezes, certeza, ou mais que ela foi internada esse ano. Duas, eu tenho certeza!* (F. 21)

*Olha, nos últimos 12 meses, ela deve ter sido internada em umas cinco, seis vezes. Internada entre aspas, porque, tipo assim, que ela foi pro hospital, porque às vezes ela ficou lá, passou o dia no pronto-socorro e voltou pra casa, entendeu? Internada mesmo, a longo prazo, foram três vezes.* (F. 25)

Sob outro ângulo, observaram que o planejamento da transição é um processo ativo e que, por mais que uma previsão de alta tenha sido orientada, as avaliações diárias traziam mudanças.

*Que as duas bolsas de sangue e aí ela melhorando, o doutor achou por bem da alta. Até porque ficar muito tempo em hospital, como ela já é de idade, e tem outras comorbidades, é um risco também que corre.* (F. 13)

*Primeira vez, soube que era com sete dias* [período de internação]. *Como foi melhorando durante o tratamento, foi melhorando, e o doutor falou: “Não é com três dias”. E eu fui internada quarta-feira e saí sexta-feira.* (P. 24)

### Condições facilitadoras e inibidoras da transição do cuidado

Alguns fatores foram assinalados como desafios à transição da alta, em especial a dinâmica familiar e a renda. A Figura 3 traz esses dados.

**Figura 3:** Tabela falante das condições facilitadoras e inibidoras das transições dos pacientes que tiveram alta da clínica médica ou cirúrgica, Coxim, Mato Grosso do Sul, Brasil, 2022

Alguns pacientes, além dos arranjos familiares de organização do cuidado durante o período de convalescência, tiveram que transitar pelo domicílio de seus familiares e amigos.

*Para ela, fica melhor eu aqui. Eu lá, e ela aqui fica mais difícil. E eu estando aqui pra ela é mais fácil. Na hora que eu melhorar bem, eu vou pro meu ranchinho de novo.* (P. 10)

*Eu fui lá para casa da minha amiga que estava comigo, que ela não quis me deixar sozinha, porque aqui só está eu e meu irmão. O meu irmão não para. Então ela falou assim: “Vamos na sua casa, pegue suas coisas e vamos embora para minha casa”. Eu fiquei 14 dias lá com ela, até eu me recuperar um pouco para poder vir para casa.* (P. 14)

A baixa renda se destacou entre as condições inibidoras para a transição do cuidado.

*Ele tem que ter alguma ajuda do governo, porque como que ele vai pagar o aluguel, a água, a luz, como ele vai se manter aqui? E ele tá sem trabalhar, porque não tem como trabalhar.* (F. 07)

*Essa pomada é 114 reais. O Hidrogel não é uma pomada barata. Está cheia. Já foram duas.* (P. 10)

*Já tenho minha senhora que trata há muitos anos, e eu tenho que trabalhar muito, eu estou ficando descapitalizado. Eu não estou dando conta, tudo é particular, quase tudo é particular. Ela está vendo aí, durante os anos, uma tomografia computadorizada é 750, um dia 800, 900 reais, um dia 1.300, 1.200.* (F. 21)

O acompanhamento após a transição para o domicílio, pela Atenção Primária, dividiu a opinião dos participantes.

*Até hoje, ninguém veio fazer visita aqui em casa, mas essa semana eu fui lá e eles falaram que vão vir, que a partir dessa semana vem o médico com a enfermeira, mas eles não vieram.* (F. 02)

*O posto aqui demora pra marcar. Ele me deu, o doutor lá, me deu um encaminhamento pra ir pro posto, pra marcar lá.* [...] *só quem veio aqui foi a menina para trazer aquele encaminhamento* [...] *a agente de saúde, só ela. Mas ela falou que qualquer dúvida que eu não puder ir lá, elas vêm aqui, a enfermeira com a médica. Mas não chegaram ainda, não* (P. 12)

*Não. Até agora não veio ninguém* [equipe da UBS]. (P. 23)

*Ixi, é difícil de alguém passar, só a ACS.* (F. 23)

*Recebi a visita do médico, da enfermeira e da minha agente de saúde do postinho.* (P. 15)

*E a gente tem essa dignidade, esse respeito de falar, porque antes eu que vinha tratando dela, era eu, só eu e Deus, não tinha esses acompanhantes* [cuidadora]. *O médico teve aqui, acho que por duas vezes, uma vez eu não estava, ele veio aqui, veio a menina dali que trabalha, como é que fala?* [agente comunitária de saúde]. *A enfermeira, a* [técnica de enfermagem] *veio também.* (F. 21)

### Padrões de resposta da transição

Observou-se uma diminuição da média de dependência para a realização de AVDs após a alta hospitalar, quando comparada com o período que o paciente esteve internado no hospital, evoluindo de dependência moderada para dependência leve, conforme demonstrado na Figura 4.

**Figura 4:** Tabela falante dos padrões de resposta das transições dos pacientes que tiveram alta da clínica médica ou cirúrgica, Coxim, Mato Grosso do Sul, Brasil, 2022

Os pacientes e familiares associaram o processo de transição do hospital para o domicílio e evolução do quadro de saúde à habilidade de andar e realizar AVDs.

*Quando ela recebeu alta, ela não estava andando. O fisioterapeuta disse que era pra andar, mas agora já faz caminhada e tudo. Foi aí, quando ela tirou a sonda, depois que ela tirou a sonda, começou a andar.* (F. 19)

[...] *eu fiquei de pé com o andador. Daí eu dei uns dois passinhos, sozinha, comigo segurando. Daí eu comecei devagarzinho, andando de lá até aqui, depois logo comecei a largar o andador e comecei a andar. Foi assim.*[...] *vou no banheiro, ligo, desligo o ar, sento na cama, quando eu não tenho sono, limpo a casa também. Às vezes, eu assisto TV.* (P. 22)

*Ele veio de ambulância para cá, não foi fácil, porque ele não estava nem levantando nem sentando nem andando, ele estava totalmente imobilizado, nem sentava sozinho* [...] *aí, chegou aqui em casa, 11 dias depois, eu fiquei sem saber o que fazer, porque você vê uma pessoa que anda, e de repente, não está andando, não está sentando, não está fazendo nada, não está comendo, foi complicado mesmo!* (F. 02)

Outros pacientes analisaram que não estavam aptos para a alta hospitalar, e não houve manejo da dor, o que gerou queixas e desconfortos, de acordo com os relatos abaixo.

*Ela sentiu muita dor. Ela sente dor até hoje. Sexta-feira, eu levei ela no hospital pra tomar remédio, porque ela não estava suportando de dor.* (F. 10)

*Eu já sai com dor, mesmo assim eles deram alta. Voltei outra vez, aí eu fui lá, tomei o sorinho com o remédio, melhorei. Mas não pode por aquela pomada, por aquela pomada me deixa louca. Eu chego aqui, fica dor o dia inteiro, a noite inteira, mulher, eu não suporto. Como que eu vou aguentar uma dor dessa de dia e noite?* (P. 10)

*Os três dias eu não passei bem, não, foi bem ruinzinho pra mim.* [...] *Os três dias após a alta. Ela até me queria me trazer para o pronto-socorro, falei: “Não, não é necessário, deixa eu ver, se eu não melhorar, a gente vai”.* (P. 14)

*Então, eu até fiquei preocupada, sabe? Porque, tipo assim, o tempo todo, muita sonolência, você entendeu?* [...] *e daí, eles pegaram e tiraram a sonda pra ela vir embora. E daí, ela continuou o tratamento em casa. Você sabe o que é uma pessoa que dorme 24 horas? Aquela pessoa lenta, não abria os olhos, não conversava, você entendeu? Ela não estava tendo reação, né? Dormia muito o tempo todo. Tanto é que a medicação parou na quinta-feira, tá? Ontem à noite que ela começou a reagir.* (F. 25)

As narrativas dos participantes convergem com os resultados dos questionamentos abordados no CTM-15, e é possível visualizar melhor na Figura 5. A figura representa uma demonstração dos construtos basilares da teoria, organizados em quatro fatores do CTM-15, sendo: Preparação para autogerenciamento; Entendimento sobre medicações; Preferências asseguradas; e Plano de cuidado.

**Figura 5:** Tabela falante do diagrama representativo do processo das transições, Coxim, Mato Grosso do Sul, Brasil, 2022

## DISCUSSÃO

A transição do cuidado da internação para o domicílio foi influenciada por diversos fatores. Em relação à natureza das transições, sobressaíram relatos de retorno após a alta hospitalar, em que, de 65,4% participantes que buscaram por atendimento em saúde posterior à alta hospital, 41,2% referiram-se procura por atendimento de urgência. Como ilustrado nas narrativas acima, o retorno não programado ao serviço de urgência com as mesmas queixas clínicas exige a responsabilidade, desenvolvimento e implementação de um planejamento de alta eficaz, seguro e responsável. Além disso, gera um alerta epidemiológico que deve ser encarado como um indicador de desempenho dos serviços de saúde^(20,21)^.

A fim de evitar ou, ao menos, minimizar a procura por atendimento de urgência e as reinternações após a alta hospitalar, o planejamento da alta deve objetivar identificar e definir as necessidades dos indivíduos dentro do contexto que abrange a alta do hospital para a comunidade, direcionando um olhar atento à transição do cuidado. Para tanto, são necessárias práticas que tenham o propósito de aperfeiçoar o gerenciamento da alta hospitalar de forma a garantir a continuidade da assistência ao paciente^(22,23)^.

As diversas atividades exercidas pelo enfermeiro durante o plano de alta podem se distinguir entre os estabelecimentos de saúde. Tencionando desenvolver e validar uma lista de atividades a serem realizadas pelo enfermeiro na alta hospitalar, estudo metodológico alcançou a validação de 13 das 14 atividades para a alta hospitalar responsável, contribuindo para o processo de alta segura e ocasionando a melhoria da qualidade do atendimento prestado ao paciente^(24)^.

Cumpre destacar que o plano de alta implementado no serviço investigado se apresenta limitado às questões de vigilância de infecções relacionadas à assistência à saúde e, por conseguinte, não atende às necessidades de saúde do paciente, o que é reforçado pelo relato do paciente que não reconhece ter recebido o plano de cuidado. Embora o fator plano de cuidados tenha sido o melhor avaliado nesta investigação, talvez justificado pelo uso do plano de alta supracitado, nenhum dos fatores recebeu uma avaliação satisfatória com pontuação maior que 70 pontos.

Estudo de revisão com ensaios clínicos randomizados comprovou que um plano de alta estruturado e personalizado para o indivíduo proporciona uma diminuição nas taxas de reinternação não programada para pacientes idosos e, ainda, aumentam as chances de reduzir o tempo de internação, o que poderia liberar a capacidade para admissões subsequentes em um sistema em que há escassez de leitos hospitalares^(25)^. Nesse sentido, a implementação do planejamento de alta eficaz, responsável e seguro no hospital não minimizaria apenas a procura por atendimento de urgência após a alta hospitalar, mas também reduziria o tempo de internação do paciente.

A dinâmica familiar e a condição financeira são fatores que influenciaram a transição do cuidado, reforçando a importância de se conhecer a realidade socioeconômica e cultural da população que faz uso dos serviços de saúde, uma vez que 65,4% dos participantes deste estudo apresentaram uma renda familiar de até dois salários mínimos. Estudo realizado com 289 idosos em um município de médio porte do interior de São Paulo analisou o gasto privado com a compra de medicamentos, e o comprometimento da renda familiar apontou que 68,9% dos participantes tinham classificação econômica tipo C (renda média familiar R$ 1.740,00 - R$ 3.085,00), consoante ao Critério de Classificação Econômica Brasil (CCEB). Logo, em alguns casos, essas despesas tornaram-se nocivas ao orçamento familiar^(26)^.

Dentro dos fatores determinantes de saúde, a baixa renda dos participantes é uma condição inibidora do processo de transição saudável, sendo um fator agravante para o acesso e custeamento dos fármacos, como verificado nas falas dos participantes. A aquisição de medicamentos é uma das formas pelas quais pode se manifestar a desigualdade na sociedade. A ampliação da provisão gratuita de medicamentos seria necessária para expandir o acesso e evitar gastos^(26)^. Somado a isso, outro estudo corrobora a afirmação que os usuários do sistema público de saúde têm dificuldades para completar e/ou aderir ao tratamento devido à falta de medicamentos nas unidades; consequentemente, a presença do profissional farmacêutico nas Unidades de Saúde da Família pode garantir maior acesso dos usuários aos medicamentos, bem como o uso racional^(27)^. Os altos custos com a prescrição de medicamentos não contemplados pelo Sistema Único de Saúde e exames solicitados podem favorecer o abandono do tratamento, resultando em retornos ao serviço de emergência e uma transição insalubre.

A transição do cuidado tornou-se um dos domínios interligados aos princípios da integração dos sistemas de saúde^(28)^. Nessa direção, a Atenção Primária à Saúde (APS) é um recurso facilitador do processo de transição, além de ser considerada a principal estratégia de reorientação do modelo assistencial, indicada como centro de comunicação das redes, promovendo a equidade, o acesso, a qualidade da assistência em saúde e a continuidade do cuidado prestado^(29)^. No entanto, parte significativa (80,8%) dos participantes relatou que não recebeu a VD de qualquer membro da equipe de saúde da APS. Infere-se a falta de comunicação entre as redes de atenção do município, assim como um documento de contrarreferência, o que reflete uma lacuna na transição do cuidado.

A não realização de VDs foi um achado de estudo que teve como objetivo identificar a prevalência de não realização de VD pelos Agentes Comunitário de Saúde (ACS) e os fatores associados, demonstrando que 18,6% das famílias não foram visitadas. A referida investigação evidenciou que 52,0% dos usuários disseram que o ACS da área não realizava visitas à casa; 26,0% não sabiam da existência de ACS no bairro ou unidade; e 10,9% referiram que, durante o horário de trabalho do ACS, não havia ninguém em casa para atendê-los^(30)^. Nesse rumo, outro estudo abordou que as VDs são estratégias de monitoramento da saúde e facilitadoras da transição saudável, provando ser eficientes na gestão do cuidado, permitindo a criação de laços entre famílias e profissionais e aumentando as chances de um cuidado longitudinal^(28)^.

Os indicadores de processo e de resultado da transição do cuidado são padrões de resposta a serem avaliados. Os cuidados habituais prestados durante o período de internação promoveram uma diminuição da dependência para a realização de AVDs após a alta hospitalar. Tal dado sugere que, no decorrer do período de hospitalização dos participantes, os cuidados prestados auxiliam o retorno da autonomia nas AVDs pós-alta hospitalar, e essa é uma importante questão a ser observada no decurso da hospitalização e no preparo para a alta do setor de internação para o domicílio.

Outro padrão de resposta a ser observado é o desempenho da transição. A média de escore do CTM-15 neste estudo foi de 59,2 pontos, distante de ser considerado satisfatório. Estudo que utilizou o CTM-15 no sul do Brasil contou com a participação de 117 pacientes e 81 cuidadores, e avaliou a qualidade da transição do cuidado de pacientes com doenças crônicas não transmissíveis na alta do serviço de emergência para o domicílio, por meio de contato telefônico. A média de escore do CTM-15 foi de 69,5 pontos, indicando uma qualidade moderada^(4)^.

Espera-se que a qualidade da transição do serviço de internação seja melhor ao compará-la ao serviço de emergência, visto que o tempo de permanência do paciente é maior, o que propicia aos profissionais dos setores de internação mais oportunidades de preparar o paciente para alta^(4)^. Todavia, o resultado do CTM-15 obtido neste estudo, em especial, confrontado com entrevistas presenciais em domicílio, trouxe melhor compreensão dos fatores que dificultam o alcance de padrões de resposta adequados, salientando a desconsideração da renda e condições socioeconômicas, ausência de acompanhamento nos outros níveis de atenção, dificuldade no entendimento das orientações e sentimento de não prontidão para a alta.

Nesse cenário de múltiplas necessidades e, muitas vezes, de recursos escassos, o enfermeiro exerce papel central para garantir a continuidade do tratamento e a segurança do paciente no autocuidado, liderando e coordenando esse processo. Dessa forma, atua como mediador nas ações interprofissionais, atentando para as carências e preocupações do paciente/família^(22,24)^.

A enfermagem é uma profissão independente para implementar e executar intervenções acerca do processo de cuidar. Logo, o enfermeiro, desde sua formação, é instrumentalizado para realizar ações de educação em saúde e prescrições de cuidados que fazem parte do planejamento de alta segura. Por sua vez, o planejamento de alta hospitalar é de responsabilidade da equipe multiprofissional, contudo o enfermeiro tem papel fundamental na identificação das necessidades do paciente, na sua educação, de seus familiares e na articulação com a Rede de Atenção à Saúde, fortalecendo a continuidade do cuidado após a alta hospitalar^(7)^.

Enfermeiros e a equipe multidisciplinar devem se atentar para ações que comtemplem um planejamento de alta eficaz, seguro e responsável, de forma a minimizar a busca por serviços de urgência logo após a alta hospitalar e promovam melhores resultados para a recuperação do bem-estar e qualidade de vida após a alta.

### Limitações do estudo

A principal limitação está relacionada ao tipo do participante, uma vez que o estudo propôs analisar a transição do cuidado de pacientes em internação hospitalar para o domicílio, e levou-se em consideração a perspectiva de paciente e familiares, excluindo os profissionais, que são essenciais dentro do processo de transição. Portanto, sugere-se que estudos posteriores ampliem para os demais integrantes envolvidos no processo de transição, com maior número amostral de pacientes e familiares.

### Contribuições para a área da enfermagem

O estudo contribui para a enfermagem, ao analisar o desempenho da transição dos cuidados do setor de internação para o domicilio, de acordo com a experiência de pacientes e familiares, sugerindo uma revisão/reflexão do planejamento de alta instituído pelos estabelecimentos hospitalares, de forma a subsidiar a implementação para um melhor desempenho das ações voltadas para o planejamento da alta hospitalar.

## CONCLUSÕES

A análise da transição do cuidado, utilizando-se o construto teórico de Meleis, evidenciou que a transição do hospital para o domicílio está distante de ser considerada satisfatória. Tal interpretação perpassa diversos fatores, entre eles: a condição socioeconômica e cultural dos pacientes e seus familiares; a falta de comunicação com a Rede de Atenção à Saúde; o sentimento de não prontidão para alta por parte dos pacientes e familiares; e a dificuldade de compreender orientações prestadas pela equipe. Estes fatores interferem diretamente no alcance de uma transição satisfatória.
